# Ovarian clear cell cancer associated with Trousseau syndrome: A case report and literature review

**DOI:** 10.1097/MD.0000000000032106

**Published:** 2022-11-25

**Authors:** Yilin You, Xing Chen, Yi Jiang, Wenjun Cheng

**Affiliations:** a Department of Gynecology, the First Affiliated Hospital with Nanjing Medical University, Nanjing, China.

**Keywords:** cerebral infarction, ovarian clear cell cancer, Trousseau syndrome

## Abstract

Trousseau syndrome is known as patients with tumors have a combination of multiple thrombi with systemic organ infarction. The incidence of ovarian clear cell cancer associated with Trousseau syndrome is relatively low. We report the case of a 41-year-old woman diagnosed with ovarian clear cell cancer with deep vein thrombosis and cerebral infarction who had a poor prognosis.

**Patient concerns:**

A 41-year-old woman was brought to our hospital with abdominal pain. Abdominal computerized tomography scan suggested large mass of ovarian origin which was considered an ovarian tumor with pelvic metastasis and peritoneal metastasis. Laboratory analyses indicated an elevated levels of serum tumor marker carbohydrate antigen 125 was 321.9 U/mL and the level of D-dimer was 16.71 mg/L.

**Diagnosis:**

The patient underwent pelvic mass aspiration was diagnosed with ovarian clear cell cancer. B-ultrasound revealed thrombosis of the lower limbs.

**Interventions:**

She underwent 2 neoadjuvant chemotherapies, along with anticoagulation therapy. However, it had a poor therapeutic effect, and the patient suffered from acute cerebral infarction that worsened.

**Outcomes:**

Chemotherapy and anticoagulation failed to stop the tumor and blood clot progression. The patient died 2 months after cerebral infarction without surgical treatment.

**Lessons:**

Gynecologists should be aware of the need for clinical suspicion of the risk of thrombosis during the treatment period of ovarian cancer and make careful decisions

## 1. Introduction

The concept of Trousseau syndrome (TS) was first introduced by Arman Trousseau in 1865, who described the association between malignant diseases and thromboembolic events. The clinical presentation of Trousseau syndrome is described as venous thromboembolism (VTE), including deep vein thrombosis (DVT) and pulmonary embolism, chronic disseminated intravascular coagulation associated with non-bacterial infectious thrombotic endocarditis and arterial thrombosis.^[1]^ Ovarian cancer is a malignant tumor with a high risk of VTE, according to the national data of Mulder et al in Denmark, the malignancies with higher 6-month cumulative incidence of VTE were pancreatic cancer (4.4%), ovarian cancer (3.1%), Hodgkin lymphoma (2.9%) and non-Hodgkin lymphoma (2.7%).^[2]^ Among ovarian cancer, ovarian clear cell cancer (OCCC) is a distinct entity of epithelial ovarian cancer which has shown strong relevance to thromboembolism, and its prognosis was reported to be worse than that of prevalent histologic subtypes, such as high-grade serous carcinoma.^[3,4]^

We encountered a case of patient with OCCC with TS that presented with DVT and multiple cerebral infarctions during neoadjuvant chemotherapy. Due to the special clinical significance, it deserves our attention. The patient provided informed consent, and patient anonymity was preserved.

## 2. Case presentation

**Figure 1.**
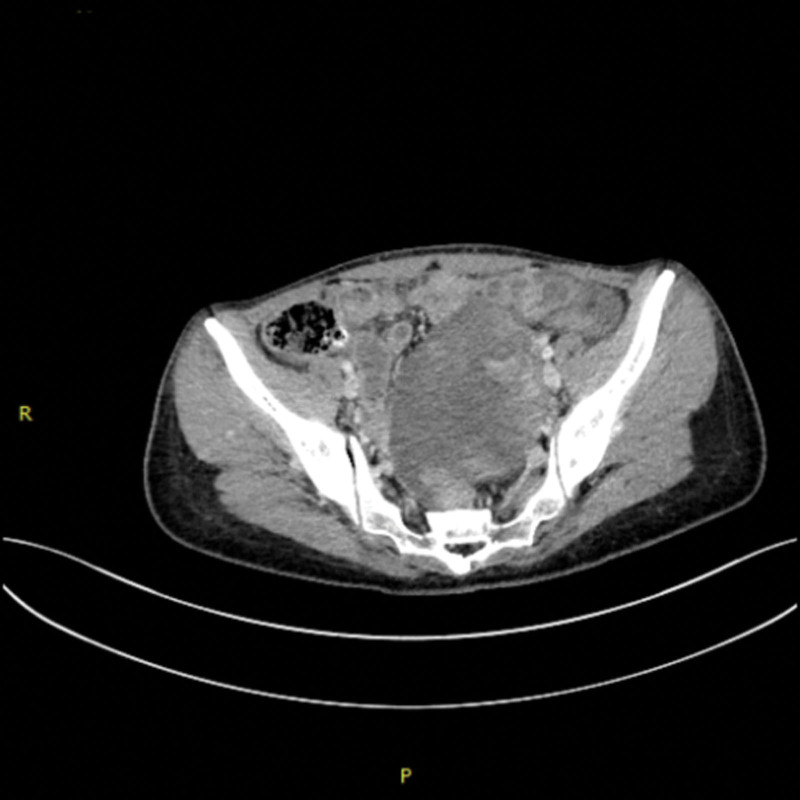
Computerized tomography (CT) scan of the abdomen. Multiple pelvic masses which were suspected metastases of peritoneal was shown.

**Figure 2.**
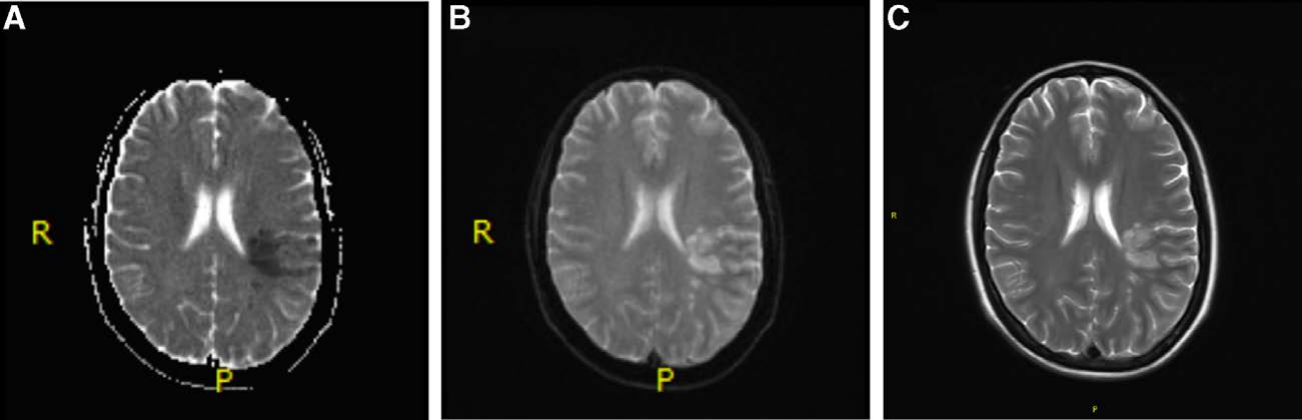
Brain magnetic resonance imaging showed cerebral infarction in the left parietal temporal lobe (A: Apparent Diffusion Coefficient sequence, B: Diffusion weighted imaging, C: T2 weighted imaging).

A 41-year-old woman presented to our hospital for abdominal pain on August 12, 2020, she had not underlying disease before. The gynecological examination results revealed a large pelvic mass with poor mobility and tenderness. Abdominal computerized tomography revealed a large mass of ovarian origin considered ovarian tumor with pelvic metastases and peritoneal metastases (Fig. 1) on August 15. Laboratory analyses indicated an elevated levels of serum tumor marker carbohydrate antigen 125 (CA125) was 321.9 U/mL (normal range: 0–35 U/mL) and D-dimer level was 16.71 mg/L (normal range: 0–0.5 mg/L). She was supposed to undergo surgery because of pelvic mass, but an unexpected fever and venous thrombosis of the lower limbs blocked the surgery. On September 1, she felt unbearable sharp pain and severe swelling of the left leg. Venous ultrasonography of the lower extremity suggested thrombosis of the left common femoral vein, superficial femoral vein, popliteal vein, and distal deep vein. Considering the high risk of perioperative thrombosis and bleeding, prophylactic anticoagulation with low molecular heparin was used. Patient was administrated with low molecular weight heparin 4000 AxaIU per day to against thrombosis. To clarify the pathology of the mass, pelvic mass aspiration was performed on September 8, and the postoperative pathology indicated clear cell cancer of the ovary. Considering the risk of thrombus dislodgement and bleeding during surgery, a primary debulking surgery could not be performed. To delay disease progression, the patient received paclitaxel 270 mg and carboplatin 450 mg on September 19 and October 10, 2020. During neoadjuvant chemotherapy, she was taking oral anticoagulants rivaroxaban 20 mg per day. Initially the level of D-dimer level slowly decreased to 3.3 mg/L and rechecked venous ultrasonography of the lower extremity showed partial recanalization on October 15. Unfortunately, she suddenly appeared aphasic, confused, and had difficulty understanding speech after the second time of chemotherapy on 30th October. Upon examination, her Glasgow Coma Scale score was 11 (E3, V3, and M5). Brain magnetic resonance imaging showed acute cerebral infarction in the center of the bilateral plate oval and left parietal temporal lobe (Fig. 2). The level of D-dimer raised to 21.55 mg/L, carbohydrate antigen 125 level did not have significant descent, which was 298.6 U/mL. Electrocardiography showed sinus rhythm and echocardiogram showed no evidence of cardiogenic embolism. She received low molecular heparin 6150 AxaIU q12h, the level of D-dimer level decreased to 6.03 mg/L, but thrombotic symptoms did not improve, the patient still had memory loss, expression difficulties, and unresponsiveness. It was difficult to decide to perform invasive surgery in such a poor condition. After careful consideration, her relatives decided to forgo chemotherapy and subsequent surgery. After discharge, the patient was treated with oral rivaroxaban 20 mg for anticoagulation, nevertheless, which did not prevent the disease from worsening. The patient died 2 months later because of multiple organ failure.

## 3. Discussion

Patients with OCCC which is a special pathological type of epithelial ovarian cancer have about a 2.5 to 4 times greater risk of developing a VTE compared than other ovarian cancer.^[5]^ Trousseaus syndrome which manifests as cerebral infarction in ovarian cancer occurs with relatively low incidence. The probability of ovarian cancer combined with cerebral infarction is approximately 1.1% to 1.7%.^[6]^ Patients with OCCC with VTE during primary cancer treatment had a shorter progression free survival and overall survival than patients without VTE. Thrombotic events are the second leading cause of death in cancer patients, after cancer itself.^[7]^ Therefore, it is important to recognize the association between OCCC and the development of TS.

The pathogenesis of arterial cerebral infarction in patients with tumors may include the following possibilities. First, the non-bacterial infectious thrombotic endocarditis caused by fibrin thrombus deposition on the surface of normal or superficially deformed heart valves is the most common cause for cerebral infraction.^[8]^ Second, tumor-associated hypercoagulability is associated not only with venous thromboembolic events, but also with cerebral infarction, that is cerebral infarction due to blood clotting in intracranial arteries. Sometimes the symptoms of thrombosis like cerebral infarction may be the first clinical sign of patients with tumor who do not have obvious signs of tumor.

The biological mechanism of high incidence of thromboembolism in patients with OCCC is complicated. Stenosis of the iliac vein due to ovarian tumor compression may be related to the DVT in our patient. OCCC specifically shows high expression of tissue factors and interleukin-6, which play critical roles in cancer-associated hypercoagulation.^[9,10]^ Tissue factors have been reported to form a complex with the coagulation factor VII to enhance the causative events in cancer progression by activating the protease-activated receptors in ovarian cancer.^[11]^ The endometriosis tumor microenvironment is characterized by chronic inflammation and hypoxic conditions, and previous studies have shown that endometriosis patients are in a hypercoagulable state like OCCC.^[9,12]^ Otherwise, angiogenesis, immune escape and metastasis facilitate tumor development, and all these processes involve activation of the coagulation system. Coagulation factors can participate in tumor progression through coagulation-dependent pathways (including fibrin formation, platelet recruitment, immunomodulatory responses, and tissue factor particle secretion) and non-dependent pathways (intracellular signaling).^[13]^

Effective treatment of thromboembolism can reduce morbidity and mortality and improves patients’ quality of life. The primary approach for treating TS is to eliminate the causative tumor through cytoreductive surgery and chemotherapy.^[6,9]^ Considering patients with a poor general condition due to TS are unable to receive effective treatment for ovarian cancer. Whether OCCC patients with cerebral infarction benefit from the standard treatment is unclear. Therefore, seeking an effective treatment is very essential. We tried to seek surgical opportunities through neoadjuvant chemotherapy, however, in this case neoadjuvant chemotherapy did not delay disease progression as we expected. The possible reason may associate with OCCC showing resistance to conventional chemotherapy and antineoplastic chemotherapy may increase the risk of vascular events. Previous reports have shown that early-onset VTE events are associated with surgery and chemotherapy during treatment of malignant diseases.^[14]^ But, Chavan et al revealed that neoadjuvant chemotherapy was not associated with thromboembolism, and the current combination of commonly used chemotherapeutic agents for ovarian cancer had a minimal risk of thromboembolic events.^[15]^ So how to balance the advantages of neoadjuvant chemotherapy and surgery in OCCC with cerebral infarction and the risks associated with treatment remains to be explored.

Low molecular weight heparin is effective for the treatment for thromboembolism in TS, currently recommended as the treatment of choice by international guidelines.^[16]^ Low molecular weight heparin was associated with less major bleeding complications compared with unfractionated heparin.^[17]^ Nevertheless, using anticoagulation therapy alone does not fully resolve the hypercoagulable state in these patients and that venous and arterial thromboembolic events may recur despite using adequate anticoagulation therapy.^[18]^ Hiroharu Kobayashi et al reported a case of mechanical thrombectomy with the potential to significantly improve the quality of life of patients with end-stage large vessel occlusion associated with TS, even though with no improvement in survival time.^[19]^ This treatment methods face the risk of high surgical risks, high costs, and recurrence of postoperative cerebral infarction, so whether it can be promoted depends on the patient’s specific situation.^[17,20,21]^ Therefore, there is an urgent need to explore the role of antithrombotic agents in ovarian cancer-related infarction, particularly, OCCC. Currently some scholars focus on targeted therapy, like IL-6-targeting therapy and Tissue Factor-targeting therapy, immunotherapy that may be the possible treatment candidate for patients with OCCC complicated with TS in the future.^[9]^

## 4. Conclusion

In conclusion, ovarian clear cell cancer is prone to a hypercoagulable state, which should be highly valued clinically. Treating TS is clinically relevant because of the presence of Trousseau syndrome, which indicates an aggressive tumor and a poor prognosis. Decision-making in terminal care is very delicate and difficult, gynecologists and neurologists jointly evaluate the condition and develop an appropriate treatment plan for the patient to balance the prognosis and quality of life. Different treatment strategies are urgently required to improve survival outcomes in this disease subset.

## Author contributions

YY wrote the article. XC and YJ contributed to the data collection and analysis. XC and WC contributed to the data interpretation and critical revision. All authors have read and approved this article.

**Conceptualization:** Yilin You, Xing Chen.

**Data curation:** Xing Chen, Yi Jiang.

**Formal analysis:** Xing Chen, Yi Jiang.

**Supervision:** Xing Chen, Wenjun Cheng.

**Writing – original draft:** Yilin You.

**Writing – review & editing:** Xing Chen, Wenjun Cheng.
